# The impact of advancing the standard of care in radiotherapy on operational treatment resources

**DOI:** 10.1002/acm2.14363

**Published:** 2024-04-18

**Authors:** Michael Roumeliotis, Kundan Thind, Hali Morrison, Ben Burke, Kevin Martell, Lukas van Dyke, Lisa Barbera, Sarah Quirk

**Affiliations:** ^1^ Department of Radiation Oncology and Molecular Radiation Sciences Johns Hopkins University Baltimore Maryland USA; ^2^ Henry Ford Cancer Institute Detroit Michigan USA; ^3^ Department of Oncology University of Calgary Calgary Alberta Canada; ^4^ University of Alberta Edmonton Alberta Canada; ^5^ Tom Baker Cancer Centre Calgary Alberta Canada; ^6^ Department of Radiation Oncology Brigham and Women's Hospital Dana‐Farber Cancer Institute, and Harvard Medical School Boston Massachusetts USA

**Keywords:** External beam, Hypofractionation, Operational resources

## Abstract

**Purpose:**

To demonstrate the impact of implementing hypofractionated prescription regimens and advanced treatment techniques on institutional operational hours and radiotherapy personnel resources in a multi‐institutional setting. The study may be used to describe the impact of advancing the standard of care with modern radiotherapy techniques on patient and staff resources.

**Methods:**

This study uses radiation therapy data extracted from the radiotherapy information system from two tertiary care, university‐affiliated cancer centers from 2012 to 2021. Across all patients in the analysis, the average fraction number for curative and palliative patients was reported each year in the decade. Also, the institutional operational treatment hours are reported for both centers. A sub‐analysis for curative intent breast and lung radiotherapy patients was performed to contextualize the impact of changes to imaging, motion management, and treatment technique.

**Results:**

From 2012 to 2021, Center 1 had 42 214 patient plans and Center 2 had 43 252 patient plans included in the analysis. Averaged over both centers across the decade, the average fraction number per patient decreased from 6.9 to 5.2 (25%) and 21.8 to 17.2 (21%) for palliative and curative patients, respectively. The operational treatment hours for both institutions increased from 8 h 15 min to 9 h 45 min (18%), despite a patient population increase of 45%.

**Conclusion:**

The clinical implementation of hypofractionated treatment regimens has successfully reduced the radiotherapy workload and operational treatment hours required to treat patients. This analysis describes the impact of changes to the standard of care on institutional resources.

## 1 INTRODUCTION

Clinical trials in radiotherapy have emphasized a shift to shorter fractionation regimens by leveraging more conformal radiation delivery, careful patient selection, and better radiobiological modeling. This has been accomplished by improving radiotherapy technology and maturing knowledge of the appropriate target volumes.

Standard fractionation regimens are still commonly used in specific disease sites, though substantial advancements have been made to validate hypofractionated regimens in many site groups, with notable successes in breast,^1^, ^2^, ^3^, ^4^, ^5^ prostate,^6^, ^7^, ^8^ lung,^9^, ^10^ and central nervous system (CNS).^11^, ^12^, ^13^ Worldwide, institutions have implemented these trial protocols and have undoubtedly impacted the resources required to deliver radiotherapy effectively. The resources required for tasks such as volume contouring, treatment planning, and quality verification have been reported for radiotherapy professionals in the pre‐treatment setting. These previous studies have demonstrated a measurable increase in human capital requirements due to advanced technological integration, such as advanced imaging and stereotactic treatment requirements.^14^

The effect of integrating hypofractionated regimens has been previously reported, focusing on reducing the average number of fractions required for a patient undergoing radiotherapy treatment.^15^ The impact of hypofractionated regimens on the linear accelerator utilization and radiation therapist workload required to deliver an external beam radiotherapy course is not fully described by only the number of fractions required to treat patients. Hypofractionation regimens often require more advanced verification imaging,^16^, ^17^, ^18^ treatment protocols that include challenging bladder and rectal filling specifications,^19^, ^20^ respiratory motion management,^21^, ^22^, ^23^, ^24^ adaptive radiotherapy workflows,^25^, ^26^, ^27^ or more advanced immobilization^28^, ^29^ than the previous treatment standards. These technological advancements alter the setup, immobilization, imaging, and treatment time per fraction, which may impact the operational treatment resources required to treat patients in external beam radiotherapy.

This study reports external beam radiotherapy fractionation trends and linear accelerator operational treatment hours across a decade in a large multi‐institutional setting. These trends are contextualized by sub‐analyses of fractionation changes for curative and palliative regimens and select examples in disease site groups where the standard of care has changed significantly in the decade. While institutional experience will vary, the core influencing factors across institutions are similar. The analysis of treatment time and operational hours is combined with an analysis of treatment preparation resources as measured by medical dosimetrist, radiation oncologist, and medical physicist task time. This study is a quantitative report that provides the context of the linear accelerator workload impact of hypofractionation prescription regimens, which offsets the increased treatment time associated with more complex technologies and techniques. It is imperative for the radiotherapy community to understand better the changing resource requirements associated with the implementation of new treatment regimens.

## 2 METHODS

### 2.1 Overview

This study uses radiation therapy data from two independent tertiary care, university‐affiliated cancer centers. All treated patient plans approved and delivered between 2012 and 2021 were included. Both centers use a single vendor solution (Varian Medical Systems, Palo Alto, CA, USA) to deliver external beam radiation therapy, including ARIA RO as the Oncology Information System, Eclipse as the treatment planning system, and Varian linear accelerators. Plan descriptors were compiled by extracting data elements using the Eclipse Scripting Application Programming Interface (ESAPI) and direct database query.^30^ These descriptors include course and plan names that follow institutional naming conventions, course intent (palliative or curative), plan type, prescribed dose, and total number of fractions. Plan type includes 3D conformal radiotherapy (3DCRT), intensity modulated radiotherapy (IMRT), and volumetric modulated radiotherapy (VMAT).

The treatment time per fraction was measured from treatment records in ARIA RO as the time the radiation therapist loaded the patient plan at the linear accelerator console to the time the fraction was acknowledged as completed and electronic patient encounter closed. Summary statistics for each patient plan over their radiation treatment course were extracted from the ARIA database including mean, median, maximum, minimum, 25th and 75th quartiles. Average mean treatment times were calculated across all delivered fractions for each year. Very short (less than 3 min) and very long (greater than 300 min) were excluded as they represent incorrectly loaded and or unloaded plans. The median and range of the difference between annual overall mean scheduled and mean actual treatment time is reported. The on‐treatment resources (minutes) metric is defined as the average fractions per patient multiplied by the average treatment time per fraction. To measure the time required to prepare a patient for treatment, the task time was analyzed for all tasks completed by the medical dosimetrist, radiation oncologist, and medical physicist prior to treatment. Treatment preparation resources and on‐treatment resources are combined to provide an assessment of total treatment resources.

This study was determined to be of minimal risk and consistent with a quality improvement project using the Alberta Research Ethics Community Consensus Initiative (ARECCI) screening tool provided by the Heath Research Ethics Board of Alberta and did not require further ethics board approval.^31^

### 2.2 Curative and palliative fractionation

For each year, the average number of fractions per patient plan was calculated and further categorized by palliative and curative intent. This was reported separately for each institution and the average of both institutions combined. Boost prescriptions were excluded from the data set for average fraction number analysis to avoid conflating typical boost fraction numbers with the introduction of hypofractionation. Boost treatments were included in all time‐based analyses.

### 2.3 Operational treatment hours

For all linear accelerators, the operational treatment hours were extracted using SQL database query from the ARIA RO Appointment Scheduling data (Varian Medical Systems, Palo Alto, CA, USA) each week in the study time frame. The median for each linear accelerator for each week was summed over all 52 weeks in the year to yield the median and interquartile range for weekly operational hours annually. Pre‐treatment patient‐specific QA appointments were excluded for this analysis. For each institution's dataset, a linear regression was performed to represent the average change in treatment time per year across the decade. Radiation therapy technologist staffing full‐time equivalence (FTE) is also reported for this time frame.

Center 1 maintained a consistent number of operational linear accelerators (nine) over the decade. Center 2 increased capacity by one linear accelerator (seven to eight) in 2015. Treatment unit replacements in this time frame were completed at both institutions.

### 2.4 Tumor site group analysis: Breast and lung radiotherapy

Further analysis of the curative intent plans for Center 1 was performed on breast and lung radiotherapy cohorts to provide specific context on the impact of technological innovations such as imaging, motion management, and the transition of advanced techniques to the standard of care on treatment time. Plans included in each tumor site group were identified through institutional naming of course and plan name. This analysis intends to describe the representative influencing factors on treatment resources that may be considered when applied to an arbitrary institution. The average number of fractions and the average treatment time are reported for the breast site group. The on‐treatment resources (minutes) metric is also reported. A similar analysis is performed for the lung site group but with emphasis on the percentage of patients treated in stereotactic body radiotherapy (SBRT) to capture a representation of the changing treatment complexity coupled with hypofractionated regimens.

### 2.5 Total treatment resources

The total treatment resources for a patient were analyzed by combining the treatment preparation resources with the on‐treatment resources. A previous analysis determined the median total treatment preparation time required for all role groups for each plan category (3DCRT, IMRT, VMAT). For each year, these times were applied for each plan category to provide treatment preparation times. For this final analysis, it was assumed that two radiation therapists were required to treat each patient.

## 3 RESULTS

### 3.1 Curative and palliative fractionation

Between 2012 and 2021, the dataset includes 42 214 patient plans treated for Center 1 and 43 252 for Center 2. Across all years, less than 0.1% of patient fractions were excluded when categorized as too short (less than 3 min) or too long (greater than 300 min). The median (range) difference between scheduled and actual fractional treatment duration for delivered plans was 0.5 (−1.5 to 2.1) min. During the same period, these institutions reported an increase in patient plans per year of approximately 45%^14^ from 3200 in 2012 to 4700 in 2020.

**FIGURE 1:**
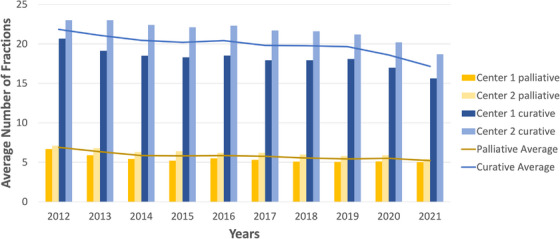
The average number of fractions for palliative (yellow) and curative (blue) intent plans are displayed for two institutions. The lines indicate the average between the two centers.

For both institutions, the average number of fractions for palliative and curative intent patient plans is displayed in Figure 1. There is a consistent trend in reducing the average fraction number of palliative and curative patients. For curative patients (blue), the average fraction decrease is 21% across the decade. For palliative patients (yellow), similar trends are reported for both centers, showing a decrease of 25% in average fractions across the decade.

### 3.2 Operational treatment hours

**FIGURE 2:**
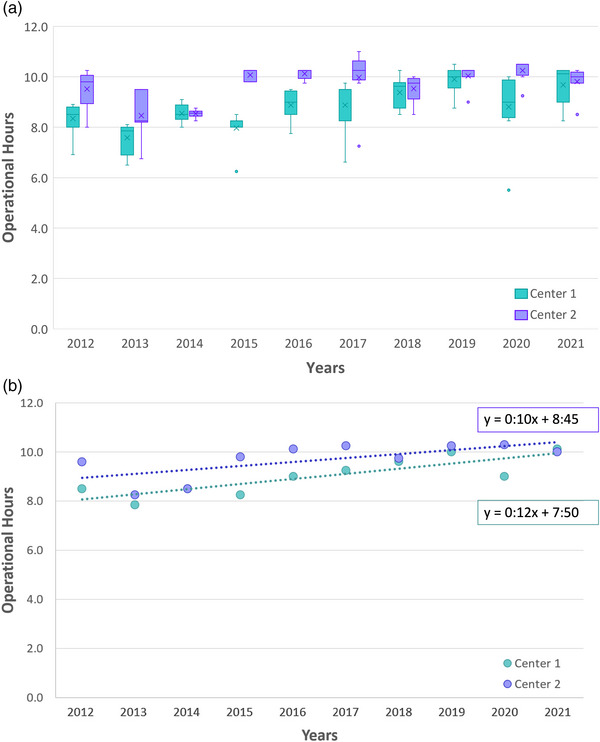
Institutional operational hours. (a) shows both centers' median operational hours and interquartile range across the decade. (b) shows the median operational hours with a linear regression trendline, where the slope indicates the expected increase in operational hours (minutes) per year. For each box and whisker entry, the cross is the mean, the horizontal line is the median, and outliers are shown with circles.

For each center's annual operational treatment hours, the median and interquartile range are displayed in boxplots (Figure 2a) and the linear regression trendline to the median (Figure 2b). For both centers, the average increase in operational hours (18%) is small compared to the larger increase reported in the patient population (45%). The increase in operational hours was 10 and 12 min per year for Center 1 and Center 2, respectively. Had operational hours increased with patient throughput with no changes in treatment regimens or complexity, the expected median weekly operational hours would be approximately a 12‐ to 14‐h treatment day (14). The radiation therapy technologists’ FTE increased 9% between 2012 and 2021.

### 3.3 Tumor site group analysis: Breast radiotherapy

A total of 6505 patients were treated with curative intent breast radiotherapy across the decade. From 2012 to 2021, the standard fractionation regimens (95% of patients) were 42.5 Gy in 16 or 40 Gy in 15 (standard whole breast irradiation), 27 Gy in 5 (accelerated partial breast irradiation [APBI]),^32^ and 26 Gy in 5 (FAST‐Forward).^3^, ^33^, ^34^ For this breast cohort, the average fractions per treatment decreased from 17.5 in 2012 to 10.9 in 2021. These data are reported in Table 1, as well as the total number of patients per year, treatment time per fraction, on‐treatment resources, and the proportion of patients treated in deep‐inspiration breath hold (DIBH).

**TABLE 1:** Treatment characteristics for breast radiotherapy.

Year	Patients	Average fractions	Average treatment time per fraction (minutes)	On‐treatment resources (minutes)	Patients treated in DIBH
2012	537	17.5	12.1	212	0%
2013	537	17.5	13.8	242	10%
2014	516	17.4	14.9	259	38%
2015	608	17.0	13.7	233	37%
2016	627	16.1	15.6	248	33%
2017	674	15.6	17.7	276	51%
2018	706	15.4	18.2	280	45%
2019	732	16.1	18.0	290	48%
2020	730	14.3	19.6	280	46%
2021	865	10.9	19.7	215	54%

*Note*: Each year provides the total number of patients undergoing breast radiotherapy and average fractions and treatment time per fraction. On‐treatment resources (average fractions multiplied by average treatment time per fraction) are also reported. The percentage of patients treated with DIBH is provided for context.

Abbreviation: DIBH, deep inspiration breath hold.

**FIGURE 3:**
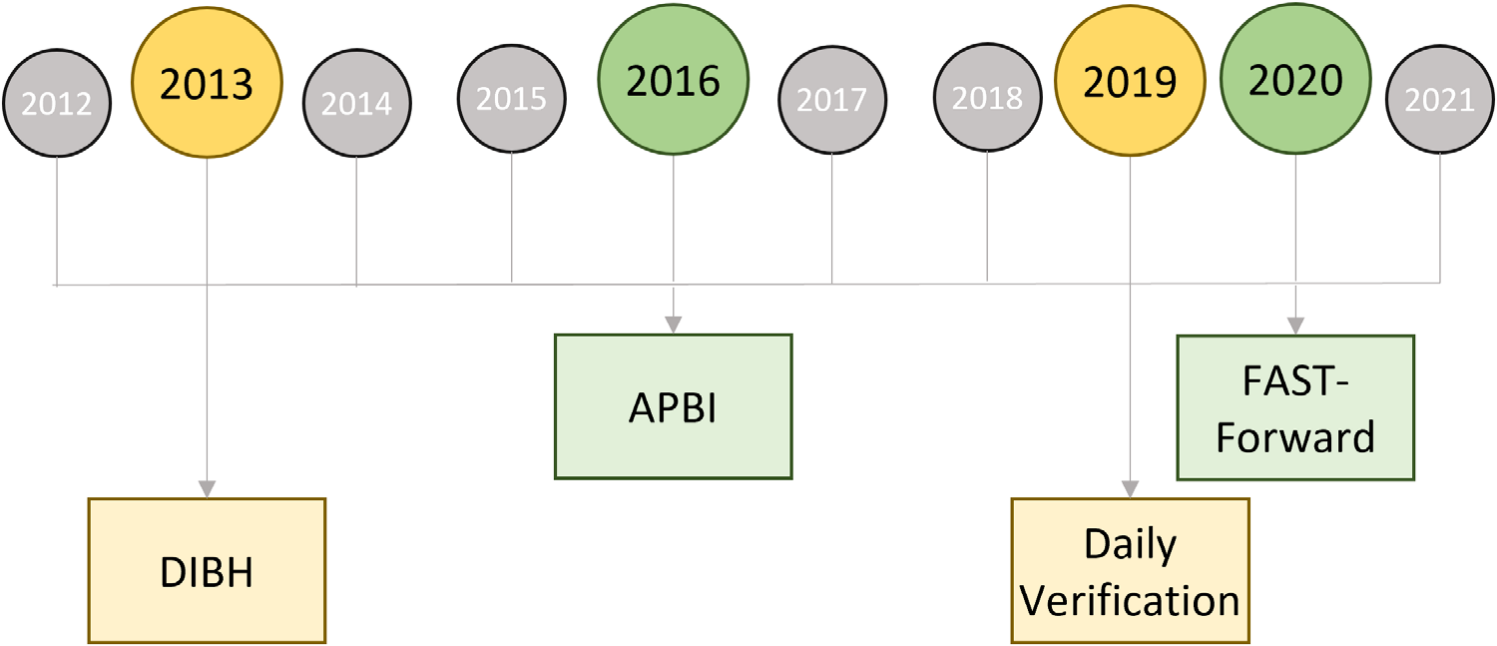
Breast radiotherapy landmark treatment changes. Major changes to prescription regimens (green) and treatment techniques (yellow) are displayed. DIBH and APBI represent a significant change to standard practice, where daily imaging replaced weekly imaging. APBI, accelerated partial breast irradiation; DIBH, deep inspiration breath hold.

The total on‐treatment resources were 212 min in 2012, increasing to a maximum of 290 min in 2019 before decreasing to 215 min in 2021. Figure 3 visually represents the timeline for implementing new prescription regimens, treatment techniques, and motion management to benchmark broad changes observed in the data reported in Table 1.

### 3.4 Tumor site group analysis; lung radiotherapy

**FIGURE 4:**
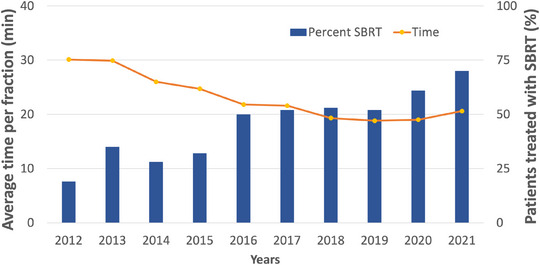
For curative intent lung, the percentage of patients treated with SBRT increased to 69.7% by 2021, and the time to deliver each SBRT fraction decreased by almost 10 min. SBRT, stereotactic body radiotherapy.

A total of 2882 lung patient plans and with 1564 curative intent were included in the analysis. Curative intent includes SBRT and radical lung treatments. The standard fractionation regimens (95% of patients) for SBRT are 48 Gy in 4, 60 Gy in 8, and 60 Gy in 15; for radical, the regimens are 60 Gy in 30 and 60 Gy in 15, which is distinguished from the same SBRT prescription by course naming convention. Across all curative intent plans, SBRT use increased from 18.3% in 2012 to 69.7% in 2021 (Figure 4). Also shown in Figure 4, there was a decrease in average treatment time per SBRT fraction from 30 min in 2012 to 20 min in 2021. These curative intent data are reported in Table 2, as well as the total number of patients per year, treatment time per fraction, on‐treatment resources, and the proportion of patients treated with SBRT. The decrease in SBRT time per fraction resulted in a decrease in on‐treatment resources, from 356 min in 2012 to 268 min in 2021. In contrast to breast radiotherapy, the substantial decreases observed per fraction treatment time are partly related to increased familiarity, standardization, and exposure to SBRT as an advanced treatment technique.

**TABLE 2:** Treatment characteristics for curative lung radiotherapy; curative intent includes stereotactic body radiotherapy and radical lung treatments.

Year	Patients	Average fractions	Average treatment time per fraction (minutes)	On‐treatment resources (minutes)	Patients treated in SBRT (%)
2012	101	16.6	21.5	356	19
2013	109	14.4	22.5	324	35
2014	135	14.4	20.6	298	28
2015	137	15.0	18.9	284	32
2016	159	13.4	18.2	244	50
2017	156	13.1	18.2	239	52
2018	164	12.9	17.2	221	53
2019	193	13.8	18.4	254	52
2020	199	12.6	19.0	239	61
2021	211	13.3	20.2	268	70

*Note*: Each year provides the total number of patients undergoing lung radiotherapy and average fractions and treatment time per fraction. On‐treatment resources (average fractions multiplied by average treatment time per fraction) are also reported. The percentage of patients treated with SBRT is provided for context.

Abbreviation: SBRT, stereotactic body radiotherapy

### 3.5 Total treatment resources

For patients in all site groups, the total treatment resources are reported in Table 3. The average total treatment resources increase substantially across the decade, with the majority of the increase being attributed to the treatment preparation tasks, including volume contouring, treatment planning, and quality assurance.

**TABLE 3:** Total treatment resources for all patients.

Year	On‐treatment resources (hours)	Treatment preparation resources (hours)	Total treatment resources per patient (hours)
2012	8.4	7.4	15.8
2013	8.3	6.7	15.0
2014	8.4	7.2	15.6
2015	8.0	8.9	16.9
2016	8.1	11.1	19.2
2017	8.4	11.6	20.0
2018	8.7	12.1	20.8
2019	8.9	12.4	21.3
2020	9.6	13.0	22.6
2021	9.3	13.4	22.7

*Note*: The change in total treatment resources is largely contained within the treatment preparation resources.

## 4 DISCUSSION

This study reports the radiotherapy treatment resources in the setting of modernizing prescription regimens and implementing advanced treatment techniques across two institutions, each averaging over 3000 new patient treatment starts per year. The analysis of institutional operational treatment hours shows an increase much less than would be observed without hypofractionated regimens decreasing the average number of patient treatments. These trends indicate that hypofractionated regimens for appropriate disease site groups successfully reduce treatment dedicated human capital required for radiotherapy treatment delivery. The sub‐analysis of breast and lung site groups contextualizes the general factors that influence total radiotherapy treatment time resource requirements. The context provided by the treatment preparation resources demonstrates that total treatment resources required per patient plan are increasing, though increase in treatment delivery time is minimal compared to pretreatment preparation time. The study may be used to describe the patient‐centered and staff resource impact of advancing the standard of care to modern radiotherapy techniques.

Institutional operational treatment hours should be considered the global metric in resources required for delivering radiotherapy treatments, which includes changes to the patient population, the fractions per patient, and treatment time per fraction. This study is intended to be used by the radiation oncology community to provide insight into their own practices that are changing due to similar influencing factors. This is critically important for future understanding of changes to practice and working with administrators, as radiotherapy funding models are typically based on patient throughput.

The trends across all curative intent treatments indicate that hypofractionated prescription regimens have been successfully implemented and made a meaningful reduction to the patient occupancy of a linear accelerator and radiation therapist treatment resources. This is most clearly observed by the rate of increasing patient throughput (45%) compared to the rate of increasing operational hours (18%) and radiation therapy technologist staffing (9%) across the decade. Prior studies have surveyed the radiotherapy community to measure the adoption of new techniques or fractionation regimens.^35^, ^36^, ^37^ This will continue to occur at varying institutional rates, where the current study may provide a reference to the expected impact of these changes.

The operational impact on pretreatment human resources has been previously investigated for this cohort. During the same time, patient numbers increased by approximately 45%, and pretreatment time‐based human resources increased by 150%.^14^ The staffing levels for these roles increased by approximately 30% between 2012 and 2021 to account for the increase in patients and time‐based required resources. Implementing advanced technology and treatment setup strategies with hypofractionated regimens requires reporting to include radiation therapist workload and overall linac operation time. As well, the tradeoff between professional resources and patient appointment convenience should be considered comprehensively when deciding to implement new techniques.

The site group analysis reported in this work highlights the impact of new prescription regimens do not directly lead to a straightforward reduction in total treatment time per patient. In breast radiotherapy, the average treatment time per fraction increases when techniques such as respiratory motion management and daily imaging are implemented. This increase is only offset by a substantial reduction in fraction number, allowing for reduced on‐treatment resources. Conversely, lung radiotherapy demonstrates a decrease in daily fractional treatment time with the increased use of SBRT. Initially, institutional protocol relied on radiation therapists seeking guidance from oncologists and physicists at the time of patient setup, leading to extended treatment times. With increased SBRT experience, workflows can improve efficiency through standardization.

Previous work analyzed and reported the impact of increasing treatment complexity on the resources dedicated to the pretreatment preparation process.^14^ This study should be considered in conjunction with the previous work, providing a more complete representation of the entire treatment process and resource requirements for all radiotherapy professionals. Radiation therapists predominantly experience the change to personnel resources required for treatment delivery, though other radiation medicine professionals may also observe an increased workload since more advanced techniques require a greater frequency of consults, image approvals, and intervention by radiation oncologists and medical physicists. Other work has also explored the time‐based impact of treatment regimen changes to standard of care,^38^ which may be used to supplement the findings of the current study. This is aligned with editorials published by radiation oncology professional societies that have noted the challenges in generating validated data to better understand the cost of radiotherapy.^39^ To apply these findings broadly, individual institutions will assess the applicability based on their own uptake of hypofractionation and advanced treatment methods. Without an exhaustive institutional analysis, these results should motivate oncology leadership to acknowledge that there is a cost to maintaining the status quo.

The data included in this analysis is both clinical and extracted from a quantity that requires a radiation therapist to manually load the patient plan and close the patient plan. Operating hours are derived from scheduled appointments and do not account for treatment days that finish earlier or later than scheduled, though large scale trends should be captured by the operational hour reports. The comparison of scheduled and actual fraction duration times demonstrated high consistency and are on average less than a minute different. As the analysis includes all treatment machines over the entire study period, anomalous days are likely minimal impact. Total workload per machine can vary depending on the technical specifications of the unit and each does not contribute equally to total operational hours. Institutional experience may vary, but similar factors at any center will influence operational treatment hour changes beyond only the consideration of changes to the patient population.

Hypofractionation is a measurable change in practice over the last decade, which has impacted standard of care for many disease sites. There are other changes to practice that may vary by institution and are less quantifiable, such as changes in linear accelerator technology with faster mechanical operation in newer treatment units or flattening filter free (FFF) delivery that allows for a much faster dose rate and shortens beam‐on time. Previous studies have shown that there is a large reduction in percentage of beam on time with FFF delivery, but the total reduction in treatment time was less significant.^40^, ^41^

The COVID‐19 pandemic influenced clinical radiation oncology from March 2020 to the end of the study period. Changes to health and safety protocols could increase the length of patient treatments; however, the major change to practice was for COVID‐19 positive patients and that remained very low (0.5%) in both centers during the study period^42^ though may have accelerated the use of hypofractionation.^43^

The impact of new prescription regimens and treatment techniques should also consider patient resources dedicated to their own treatments, which is an important paradigm in advancing patient‐centered care. Undoubtedly, hypofractionated regimens are preferred by patients due to the reduction in hospital visits, even if offset by a marginal increase in the daily treatment times.

## 5 CONCLUSION

This study demonstrates the impact of implementing modern prescription regimens and treatment techniques on radiotherapy treatment resources. The lesser increase in clinical operational treatment hours provides confidence that introducing hypofractionation successfully reduces total clinical treatment delivery resources. As hypofractionation becomes standard, it is important for administrators to understand the nuanced relationship between decreasing the total number of fractions per patient and increased time‐based resources due to the complexity of the treatment technique. The impact of modern prescription regimens and treatment techniques should be considered a success in the shift to patient‐centered care, by substantially reducing patient treatment visits.

## AUTHOR CONTRIBUTIONS

**Michael Roumeliotis**: Conceptualization; methodology; validation; formal analysis; visualization; writing—original draft preparation. **Kundan Thind**: Conceptualization; methodology; validation; visualization; writing—review and editing. **Hali Morrison**: Methodology; formal analysis; writing—review and edit. **Kevin Martell**: Methodology; writing—review and edit. **Lukas van Dyke**: Software; formal analysis. **Lisa Barbera**: Methodology; writing—review and edit. **Sarah Quirk**: Conceptualization; methodology; validation; formal analysis; visualization; writing—original draft preparation.

## CONFLICT OF INTEREST STATEMENT

No conflict of interest directly related to the manuscript's content. S. Quirk is the PI of a Canadian Cancer Society grant on data transformation with M. Roumeliotis, K. Thind, L. Barbera, K. Martell, and H. Morrison as co‐investigators. S. Quirk and K. Thind received honorariums to present in Varian's webinar series on Automation in treatment planning.
